# MicroRNA-Mediated Post-Transcriptional Regulation of Cytochrome P450s

**DOI:** 10.3390/genes17060698

**Published:** 2026-06-16

**Authors:** Qi-Hang Yu, Sohaib Shahid, Jia-Yi Wu, Lin-Yan Zhao, Fen Li, Shao-Ying Wu

**Affiliations:** 1 School of Breeding and Multiplication, Sanya Institute of Breeding and Multiplication, Hainan University, Sanya 572025, China; qihangy006@163.com (Q.-H.Y.); sohaibshahid90@hotmail.com (S.S.); 20243006288@hainanu.edu.cn (J.-Y.W.); 20243003444@hainanu.edu.cn (L.-Y.Z.); wsywsy6000@hainanu.edu.cn (S.-Y.W.); 2 School of Tropical Agriculture and Forestry, Hainan University, Danzhou 571737, China

**Keywords:** microRNA, cytochrome P450, insecticide resistance, post-transcriptional regulation, fitness cost, RNAi biopesticides

## Abstract

The rapid evolution of metabolic resistance to chemical insecticides and the adaptation to plant allelochemicals in insect pests have become major challenges in global pest management. While the overexpression of cytochrome P450 monooxygenases (P450s) is a well-recognized classic detoxification mechanism, the upstream epigenetic and post-transcriptional regulatory networks governing this process have only recently been elucidated. In this narrative review, the latest research progress on microRNAs (miRNAs) as crucial “fine-tuners” in insect detoxification networks is systematically summarized. The classic regulatory model is highlighted: the induced or constitutive downregulation of specific miRNAs relieves the translational repression of their target P450 genes, thereby contributing to metabolic resistance to major insecticide classes, including neonicotinoids, diamides, and pro-insecticides. Furthermore, the evolutionary recruitment mechanisms of conserved miRNAs in host plant adaptation are explored, and how endocrine signals, such as juvenile hormone (JH) and 20-hydroxyecdysone (20E), synergistically regulate the miRNA–P450 axis is analyzed. The “sponge effect”, wherein highly expressed P450 mRNAs act as competitive endogenous RNAs (ceRNAs) to sequester miRNAs, and the consequent physiological trade-offs (fitness costs) resulting from the prioritization of metabolic resources toward the detoxification system are comprehensively discussed. Finally, the current core methodologies for miRNA functional validation are critically evaluated, and the application potential and ecological safety prerequisites of miRNA-based nanobiopesticides for targeted and sustainable pest management are discussed. By integrating mechanistic insights with translational perspectives, this review highlights miRNA–P450 regulatory networks as key determinants of insecticide resistance evolution and as promising targets for developing more precise, environmentally compatible pest-management strategies.

## 1. Introduction

Chemical insecticides represent a critical component of global food security strategies. However, prolonged and intensive field applications have imposed substantial selection pressure on pest populations, driving the rapid adaptive evolution of metabolic resistance [1,2]. The maintenance of high-level resistance is often accompanied by considerable fitness costs, including developmental delays and reduced fecundity. Consequently, in the absence of insecticide selection pressure, resistance levels in field populations frequently show partial reversibility [3]. Furthermore, the elevated expression of resistance-associated detoxification enzymes is rarely uniform throughout the insect body; rather, it is highly concentrated in metabolic organs such as the midgut and fat body [4]. This reversibility and tissue specificity of resistance suggest that insect adaptive responses to chemical stress depend not only on fixed genomic variations but also on highly flexible epigenetic and post-transcriptional regulatory networks. Among these, microRNAs (miRNAs), which mediate post-transcriptional repression by selectively binding to the 3′-untranslated regions (3′-UTRs) of target mRNAs, are increasingly recognized as important regulators governing insect responses to environmental chemical stress [5,6].

Within the extensive metabolic detoxification system of insects, cytochrome P450 monooxygenases, owing to their broad substrate spectrum, dominate the metabolic detoxification of various mainstream insecticides, including neonicotinoids and diamides [7]. In recent years, an increasing number of transcriptomic and functional validation studies have revealed that a conserved “miRNA–P450 regulatory axis” is emerging as a frontier hotspot in the field of insecticide resistance [8,9]. For example, in the brown planthopper (*Nilaparvata lugens*), novel-m0262-5p has been confirmed to directly regulate *CYP6ER1*, thereby mediating resistance to sulfoxaflor [10]; in the whitefly (*Bemisia tabaci*), miR-1517 and miR-276-3p mediate metabolic resistance to imidacloprid and cyantraniliprole, respectively [11,12] and in the mosquito *Culex pipiens pallens*, miR-4448 significantly alters the resistance level to deltamethrin by negatively regulating *CYP4H31* [13]. These cross-species findings suggest that the recruitment of specific miRNAs to finely reshape the expression of the P450 gene family is a key adaptive strategy for insects coping with complex chemical adversities.

Although research on the “miRNA–P450 axis” in insecticide resistance has made significant strides, the current overall evidence system is still predominantly based on expression correlation analyses. While causal validation studies are gradually increasing, there remains substantial room for improvement in research depth and systemicity. For instance, some studies lack *in vitro* targeting validation via dual-luciferase reporter assays and seldom conduct *in vivo* phenotypic interventions using miRNA agomirs or antagomirs, making it difficult to establish a complete causal evidence chain [14]. Moreover, the stability of these micro-regulatory networks under complex, multiple field stresses, as well as their translational potential in the development of nucleic acid biopesticides, requires further systematic evaluation. Based on the aforementioned analyses, the latest research progress on the miRNA–P450 regulatory axis in the evolution of agricultural pest resistance is systematically outlined in this review. Standard technological pathways for transitioning mechanism validation from “correlation inference” to “causation confirmation” are explored, and the application potential of this regulatory network in future integrated pest management (IPM) and novel RNAi-based control strategies is prospectively evaluated [15,16].

## 2. Molecular Basis of miRNA-Mediated Regulation of P450s

During the response of insects to xenobiotic stress, miRNAs are not directly involved in the metabolic degradation of toxicants; instead, they act as “fine-tuners” within the gene expression network. Through precise intervention on transcripts, the expression abundance of detoxification enzymes, particularly the P450 family, a massive enzyme superfamily utilizing heme as a cofactor that plays critical roles in xenobiotic detoxification and drug metabolism, is regulated by miRNAs at specific times and in specific tissues [5,6,7,8,9].

### 2.1. Classic Mechanism of miRNA-Mediated Post-Transcriptional Repression and Its Validation Strategies

In the classic post-transcriptional regulatory model, mature miRNAs are loaded into the RNA-induced silencing complex (RISC), where base-pairing occurs primarily between the “seed region” at the 5′ end (typically nucleotides 2–8) and the 3′-untranslated region (3′-UTR) of the target mRNA [17,18]. This binding generally leads to two outcomes: direct mRNA degradation or translational repression by ribosomes. This mechanistic framework fundamentally explains why the abundance of specific miRNAs and the expression levels of target P450s often exhibit a significant negative correlation in numerous insecticide-resistant strains [9,19].

**Figure 1:**
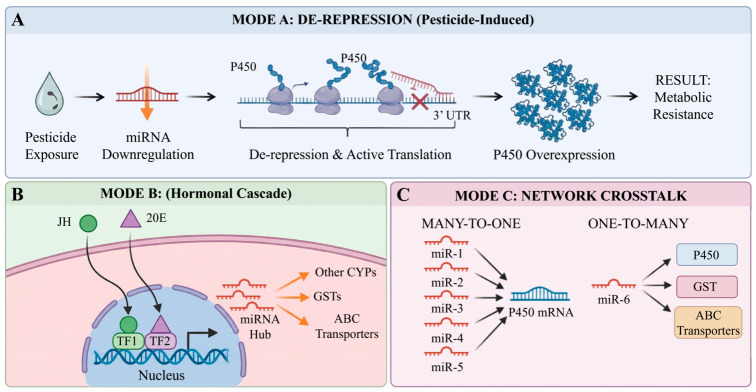
Core regulatory modes of miRNA-mediated insecticide resistance in insects. (**A**) De-repression mode: Downregulation of specific miRNAs relieves translational repression of target P450 mRNAs, leading to increased detoxification enzyme expression. (**B**) Hormonal cascade mode: Endocrine signals (juvenile hormone, JH; 20-hydroxyecdysone, 20E) regulate miRNA expression through transcription factors. (**C**) Network crosstalk mode: miRNA–target interactions include “many-to-one” regulation (multiple miRNAs targeting a single P450) and “one-to-many” regulation (a single miRNA targeting multiple detoxification genes).

In current molecular mechanism studies, a negative correlation derived solely from small RNA sequencing (sRNA-seq) is insufficient to confirm targeting relationships. Instead, dual-luciferase reporter assays combined with *in vivo* functional validation are commonly used as a more rigorous strategy to verify direct miRNA–target interactions and their functional consequences [9,14,17,18]. For instance, in studies of chlorantraniliprole resistance in the fall armyworm (*Spodoptera frugiperda*), miR-190-5p was confirmed via dual-luciferase assays to specifically bind to the 3′-UTR of its target detoxification gene *CYP6K2*, thereby exerting significant negative regulation at the post-transcriptional level (Figure 1A) [20]. Similarly, in the whitefly (*B. tabaci*), miR-276-3p was verified by the same method to mediate resistance to cyantraniliprole [12]. Beyond *in vitro* validation, *in vivo* interventions are equally critical. In studies of neonicotinoid and sulfoximine resistance in the brown planthopper (*N. lugens*), the targeting relationships between novel_85 (as well as other miRNAs) and core metabolic enzymes such as *CYP6ER1* were first confirmed *in vitro*; given that *CYP6ER1* is a key P450 gene associated with insecticide resistance in *N. lugens* and its suppression has been shown to increase insecticide susceptibility, the regulatory role of this axis on resistance phenotypes was further validated at the *in vivo* level through the injection of miRNA agomirs or antagomirs [10,21]. To improve the rigor and comparability of studies investigating miRNA–P450 interactions, it is useful to classify existing evidence into hierarchical levels based on methodological strength. At present, three major categories can be defined and are summarized schematically in Supplementary Figure S1. Level I evidence is based on expression correlation, typically derived from transcriptomic or small RNA sequencing datasets, where inverse relationships between miRNAs and P450 genes are observed [9,19]. While such analyses provide valuable screening clues, they do not establish direct regulatory interactions. Level II evidence includes *in vitro* validation, most commonly through dual-luciferase reporter assays, which confirm physical binding between miRNAs and the 3′-UTRs of target genes [17,18]. Level III evidence represents the highest level of validation and involves *in vivo* functional assays, such as miRNA agomir/antagomir manipulation or RNAi-mediated pathway disruption, coupled with phenotypic assessment of insecticide susceptibility [12,21].

Notably, although an increasing number of studies have progressed toward Level II and Level III validation, a substantial proportion of published work still relies primarily on Level I evidence [14]. Therefore, future research should prioritize integrating multi-level validation approaches to establish robust causal relationships within miRNA–P450 regulatory networks.

### 2.2. Multi-Targeting and Hierarchical Synergy of miRNA Regulatory Networks

With the deepening of mechanistic studies, the interaction between miRNAs and P450s is no longer limited to a simple “one-to-one” regulatory model but exhibits highly complex network characteristics. During long-term evolution, highly synergistic post-transcriptional regulatory networks have been constructed by insects, which are primarily manifested across multiple dimensions. For example, multiple distinct miRNAs can collectively target a single key detoxification gene (“many-to-one” regulation), and holistic regulation can be directed against detoxification gene clusters.

On the one hand, when detoxification genes undergo tandem duplication to form gene clusters, their regulatory miRNAs may also function synergistically as clusters. A typical “miRNA cluster–target gene cluster” synergistic model was discovered in the study of DDT resistance in *Drosophila melanogaster*. In the resistant strain, the miR-310s cluster (comprising miR-310, miR-311, miR-312, and miR-313) is downregulated as a whole, thereby simultaneously relieving the post-transcriptional repression of a pair of adjacent P450 detoxification genes (*Cyp6g1* and *Cyp6g2*) on the genome. This coupled effect leads to the constitutive overexpression of these two core genes, which is considered a crucial component conferring high-level DDT resistance in *D. melanogaster* [22].

On the other hand, a single miRNA may simultaneously affect different types of detoxification enzyme genes, forming crosstalk across detoxification pathways and further expanding the functional dimensionality of miRNAs in insect detoxification regulatory networks. For example, in *S. frugiperda*, miR-23a not only targets the P450 gene *CYP9F1* [23] but has also been confirmed to concurrently target the glutathione S-transferase gene *SfGSTs3* [24]. This “one-to-many” targeting mechanism enriches existing post-transcriptional regulatory theories and is considered one of the vital adaptive strategies for pests to achieve efficient detoxification and optimize transcriptional costs in complex chemical environments (Figure 1C). Collectively, these findings indicate that miRNA-mediated regulation of detoxification systems should not be viewed as a series of isolated one-to-one interactions but rather as a highly coordinated network-level control mechanism. Within this framework, miRNAs function as regulatory buffers that stabilize gene expression under fluctuating environmental stress while simultaneously enabling rapid reconfiguration of detoxification pathways [9,18]. The coexistence of “many-to-one” and “one-to-many” regulatory modes allows insects to achieve both robustness and flexibility, ensuring efficient metabolic responses to diverse chemical challenges while minimizing transcriptional and energetic costs [8].

## 3. Insecticide Stress-Driven Evolution of miRNA–P450-Mediated Resistance

Under continuous insecticide selection pressure, the upregulation of detoxification enzyme genes, particularly cytochrome P450 genes, is one of the pivotal mechanisms underlying the development of metabolic resistance in insects. Traditionally, such changes have been largely attributed to promoter mutations, transcription factor activation, and upstream signaling pathway regulation [1,2,7]. However, accumulating evidence has demonstrated that miRNA-mediated post-transcriptional regulation also plays an important role in this process [8,9]. Specifically, decreased abundance of certain miRNAs can relieve their repression on downstream P450 genes, thereby promoting the expression of related detoxification genes and enhancing insect tolerance to insecticides [10,11,12,20,21].

### 3.1. Regulatory Patterns of the miRNA–P450 Axis Under Neonicotinoid Stress

In studies on neonicotinoid resistance, the miRNA–P450 regulatory axis has been reported in various piercing-sucking pests. In the brown planthopper *(N. lugens)*, for example, *CYP6ER1* is considered one of the key metabolic genes mediating neonicotinoid resistance. In nitenpyram resistance, novel_85 binds to *CYP6ER1* and downregulates its expression, and changes in its expression significantly affect the susceptibility of *N. lugens* to nitenpyram [21]. In clothianidin resistance, miR-210-5p has also been confirmed to directly bind to *CYP6ER1*, influencing the susceptibility of *N. lugens* to the insecticide by regulating its expression [25]. These findings indicate that distinct miRNAs can converge on the same key P450 gene to form specific regulatory patterns in response to different insecticides. Analogous phenomena have been observed in other hemipteran pests. In the whitefly (*B. tabaci*), novel_miR-1517 is involved in the development of imidacloprid resistance by regulating *CYP6CM1* [11]; in the English grain aphid (*Sitobion miscanthi*), miR-3037 targets *CYP6CY2* and is closely associated with the imidacloprid resistance phenotype [26]. Collectively, these studies suggest that the “miRNA alteration–P450 de-repression–enhanced insecticide tolerance” cascade may represent a critical post-transcriptional regulatory mode by which hemipteran insects cope with neonicotinoid insecticides.

### 3.2. Rapid Metabolic Adaptation to Novel Insecticides

In addition to neonicotinoids, miRNA–P450 regulation is also involved in insect adaptation to insecticides with novel modes of action. In the fall armyworm (*S. frugiperda*), miR-190-5p and miR-23a have been reported to affect susceptibility to chlorantraniliprole by targeting *CYP6K2* and *CYP9F1*, respectively [20,23]. This demonstrates that distinct miRNAs can act independently on different P450 members to collectively mediate the metabolic response to a single insecticide. In the small brown planthopper (*Laodelphax striatellus*), miRNA PC-5p-30_205949 can simultaneously target *CYP419A1* and *ABCG23*, regulating the insect’s susceptibility to triflumezopyrim [27]. This suggests that a single miRNA may coordinate the expression of multiple resistance-associated genes under specific insecticide stress. Based on existing studies, it is evident that miRNAs, as important post-transcriptional regulators, possess considerable regulatory potential in the development of insecticide resistance; however, their specific modes of action still exhibit distinct species and insecticide specificities. These results suggest that insect adaptation to novel insecticides may not rely on the alteration of a single detoxification gene but rather involves the synergistic regulation of multiple resistance-associated genes mediated by miRNAs.

### 3.3. Dynamic Regulatory Modes of the miRNA–P450 Network

The miRNA–P450 regulatory network is not static but may exhibit different dynamic characteristics under diverse resistance backgrounds. In the DDT-resistant *D. melanogaster* strain 91-R, for example, the *miR-310s* gene cluster has been shown to bind to the 3′-UTRs of *Cyp6g1* and *Cyp6g2*, suppressing the expression of these two P450 genes through post-transcriptional regulation. Following injection of miR-310s mimics into the 91-R strain, the transcript levels of *Cyp6g1* and *Cyp6g2* decreased, accompanied by increased susceptibility to DDT. These results suggest that under long-term insecticide selection, changes in miRNA expression may be associated with the maintenance of resistance phenotypes [22].

Notably, the direction of miRNA–P450 regulation does not always manifest as de-repression of P450s. In studies of the mitochondrial pro-insecticide chlorfenapyr, miR-6497-5p in the silkworm (*Bombyx mori*) was upregulated following insecticide treatment and was found to target *CYP337A2*. This reduced the conversion of chlorfenapyr into its highly toxic active derivative, thereby improving silkworm tolerance to chlorfenapyr [28]. Because CYP337A2 is a nuclear-encoded microsomal P450 rather than a mitochondrial enzyme, this example should be interpreted as cytosolic miRNA-mediated regulation of a detoxification enzyme, rather than direct miRNA control of mitochondrial transcripts. Nevertheless, such cytosolic regulation may indirectly influence metabolic pathways that interface with mitochondrial function.

In other systems, mitochondria-associated miRNAs, also termed mitomiRs, have been shown to localize to or near mitochondria and regulate mitochondrial transcripts or nuclear-encoded mitochondrial genes [29,30]. For example, reported mitomiRs include nuclear-encoded miRNAs that translocate into the mitochondrial matrix, as well as a smaller subset that may be encoded by the mitochondrial genome itself, which can regulate intra-mitochondrial transcripts [30,31]. Whether similar mitomiR mechanisms participate in insect responses to mitochondria-targeting pro-insecticides such as chlorfenapyr remains an interesting question for future study.

Beyond agricultural pests, miRNA–P450 regulatory networks are also relevant to mosquito vectors of vector-borne diseases. In *Culex*, P450-mediated metabolism is a major mechanism of pyrethroid resistance [32], and several miRNAs have been linked to this process, including miR-4448 targeting *CYP4H31* [13], miR-279-3p regulating deltamethrin resistance through *CYP325BB1* [33], and miR-13664 targeting *CYP314A1* [34]. In *Aedes aegypti*, miRNAs have also been associated with insecticide responses, although the reported targets are not always P450s; for example, miR-33 modulates permethrin toxicity by regulating a voltage-gated sodium channel transcript [35]. By contrast, in the malaria vector Anopheles, functional evidence directly linking specific miRNAs to individual P450 genes remains limited, despite evidence implicating non-coding RNAs in pyrethroid resistance [36]. This evidence broadens the taxonomic context of miRNA–P450 regulation and highlights mosquito vectors as an important area for future investigation.

In summary, whether in the context of neonicotinoids, novel diamides, or pro-insecticides, miRNA-mediated post-transcriptional regulation represents an important regulatory layer in insecticide responses and resistance phenotypes (Table 1).

## 4. miRNA–P450 Regulatory Networks Under Plant Secondary Metabolite Stress

From an evolutionary perspective, the miRNA–P450 regulatory systems likely originated from ancestral detoxification mechanisms shaped by long-term exposure to plant secondary metabolites [2,7,40]. Phytophagous insects have historically faced complex mixtures of allelochemicals, which exerted continuous selective pressure for the development of flexible and multi-layered detoxification strategies. In this context, miRNA-mediated post-transcriptional regulation may have originally evolved as a fine-tuning mechanism to optimize metabolic efficiency and responsiveness [5,6]. Consequently, contemporary insecticide resistance can be viewed, at least in part, as an extension or reconfiguration of pre-existing regulatory modules shaped during host plant adaptation [37,38,41]. Allelochemicals produced by plants, such as alkaloids, phenols, and terpenoids, constitute continuous selective pressures that have driven the evolution of response networks in phytophagous insects, which are composed of detoxification enzymes like P450s and their upstream regulatory factors [2,40]. Consequently, detoxification regulatory modules associated with host adaptation may provide a pre-existing molecular foundation for insects to subsequently respond to chemical pesticides, thereby offering an evolutionary perspective for understanding the phenomenon of cross-resistance [2,37,38,39,40,41].

In natural ecosystems, phytophagous insects are typically exposed not to a single toxin but to the complex, simultaneous stresses imposed by multiple plant secondary metabolites. In the cotton aphid (*Aphis gossypii*), for example, Ma et al. [38] demonstrated that the transcript level of *CYP4CJ1* could be induced by both gossypol and tannic acid and that knockdown of this gene increased the sensitivity of *A. gossypii* to these two allelochemicals. This indicates the involvement of *CYP4CJ1* in cotton aphid tolerance to the relevant secondary metabolites. Further analysis revealed that the response of *CYP4CJ1* to allelochemicals involves not only transcriptional regulation but also post-transcriptional regulation mediated by miR-4133-3p. This suggests that insects may rely on multi-layered regulatory mechanisms to achieve more refined detoxification responses when coping with plant toxins.

In addition to the broad adaptation of polyphagous insects to various allelochemicals, the miRNA–P450 regulatory axis may also participate in the directional adaptation process of certain insects to specific highly toxic host plants. In the green peach aphid (*Myzus persicae*), a significant correlation between the reduced abundance of let-7 and miR-100 and the high expression of *CYP6CY3* was identified [37]. Artificial manipulation of the abundance of these two miRNAs was shown to significantly affect the tolerance of *M. persicae* to nicotine. Furthermore, after the transcript level of *CYP6CY3* was reduced by regulating the associated miRNAs, the transcript level of *CYP6CY4* was significantly elevated. This suggests that the host adaptation process involves a P450 regulatory network with certain compensatory capacities, rather than isolated changes in a single detoxification gene. These results indicate that changes in the expression of conserved miRNAs can be recruited into host adaptation-related detoxification processes, participating in the shaping of insect tolerance to specific plant secondary metabolites.

## 5. miRNA-Mediated Endocrine Signal Transduction and Fitness Costs

During the evolution of insecticide resistance in pests, high-level expression of detoxification genes is not an isolated phenotype but is closely associated with insect growth, development, and reproduction. MicroRNAs (miRNAs) serve not only as post-transcriptional regulators of detoxification enzymes such as P450s but also as molecular hubs linking upstream endocrine signals with downstream metabolic networks. In this section, the driving roles of juvenile hormone (JH) and 20-hydroxyecdysone (20E) on miRNA networks are highlighted. Furthermore, based on the competitive endogenous RNA (ceRNA) mechanism and the resource allocation model, the physiological trade-offs between metabolic detoxification and growth and reproduction under insecticide selection pressure, which ultimately generate fitness costs, are systematically analyzed.

### 5.1. Endocrine Signals

The insect life cycle and environmental adaptation are highly dependent on the synergistic control of juvenile hormone (JH) and 20-hydroxyecdysone (20E). These core endocrine signals can act as transcriptional drivers to directly regulate the expression of specific miRNAs, thereby indirectly reshaping downstream detoxification metabolic networks (Figure 1B) [41,42]. For example, in the yellow fever mosquito (*A. aegypti*), JH activates the transcription factor E75 via its receptor Met, which in turn directly activates the expression of miR-2940 to regulate development [41]. In the brown planthopper (*N. lugens*), the 20E-responsive factor BR-C represses the expression of miR-2703, thereby relieving its suppression of the chitin synthase gene *NlCHS1a* [42]. This endocrine-mediated fluctuation in miRNA expression enables insects to adjust the abundance of detoxification enzymes in tissues in real time in response to environmental pressures such as insecticide stress, while also providing a molecular prerequisite for the subsequent generation of fitness costs.

### 5.2. Physiological Trade-Offs

**Figure 2:**
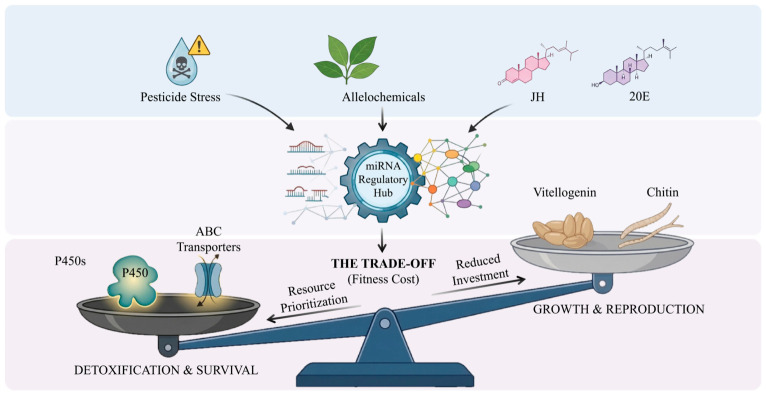
Schematic representation of miRNA-mediated physiological trade-offs in insects. Environmental stress, including insecticides and plant allelochemicals, together with endocrine signals such as JH and 20E, can reshape miRNA regulatory networks. Upregulated detoxification-related transcripts, particularly P450 mRNAs, may act as competitive endogenous RNA (ceRNA) sponges that sequester shared miRNAs. Under chemical stress, metabolic resources are preferentially allocated to detoxification and survival pathways, including P450s and ABC transporters, whereas growth- and reproduction-related processes, such as vitellogenin and chitin synthesis, receive reduced investment, ultimately resulting in fitness costs.

The acquisition of high insecticide resistance, exemplified by the massive synthesis and elevated activity of the P450 enzyme system, is often accompanied by significant fitness costs, including developmental delays (e.g., molting disorders) and decreased fecundity [3]. However, the classic “negative regulation” model of miRNAs alone cannot directly explain the apparent paradox whereby the expression of detoxification genes increases while the expression of reproductive genes (such as vitellogenin gene *vg*) decreases. At the molecular level, this phenomenon has been proposed to be associated with the sponge effect mediated by competitive endogenous RNAs (ceRNAs) [43,44]. During the evolution of resistance, detoxification genes such as *CYP6ER1* in *N. lugens* are transcriptionally upregulated [21]. The resulting abundance of P450 mRNA transcripts acts as “molecular sponges”, competitively sequestering pleiotropic miRNAs within the cell, such as miR-34-5p, which targets *Vg*, and miR-4868b, which targets glutamine synthase *(NlGS*) [45,46]. In principle, this sequestration effect could release reproduction-related target genes from post-transcriptional repression normally imposed by these miRNAs. Nevertheless, the expression levels of these genes do not increase in actual physiological phenotypes. On the one hand, the biosynthesis of the large-scale detoxification enzyme system consumes the vast majority of cellular ATP, amino acids, and protein synthesis machinery. Under extreme chemical stress, this forces the insect’s metabolic balance to tilt systematically toward survival and detoxification functions, leaving the reproductive system in a state of passive “metabolic starvation” [3]. On the other hand, reproductive genes relieved of miRNA-mediated repression are not only deprived of the metabolic resources required for their expression but are also subject to secondary downregulation by endocrine signals, such as an imbalance in the JH/20E ratio, and are susceptible to suppression by other transcriptional repressors. Under the combined effects of pleiotropic feedback repression and resource-allocation prioritization, insects pursuing survival-driven resistance may ultimately incur significant fitness costs, as schematically summarized in Figure 2.

## 6. Methodological Evaluation and Challenges

In recent years, with the rapid development of miRNA research technologies, numerous critical achievements have been made in insect miRNA research. Concurrently, faced with a series of problems triggered by the development of pest resistance, research on the involvement of miRNAs in insect pesticide resistance has increased significantly [8,9]. Early studies were mostly limited to differential expression and co-expression analyses at the transcriptome level, primarily providing correlational clues [9,14]. With the advancement of research technologies, a relatively rigorous validation paradigm for mechanism analysis in this field has been established. However, precise intervention and phenotypic confirmation at the *in vivo* level still face certain technical challenges. Importantly, different validation approaches provide distinct levels of evidence. Dual-luciferase assays confirm direct miRNA–target binding but are limited to *in vitro* systems. RNAi-based disruption of miRNA pathway components provides pathway-level insights but may introduce pleiotropic effects. In contrast, miRNA agomir/antagomir-based manipulation offers functional validation at the organismal level, although it is constrained by delivery efficiency and potential off-target effects [12,17,18,21,25]. Therefore, integrating multiple complementary approaches is essential for establishing robust causal relationships.

Nevertheless, these methodological limitations should not be interpreted as invalidating existing miRNA–P450 studies, but rather as highlighting the need for a graded evidence framework. Expression correlation analyses remain valuable for identifying candidate miRNA–P450 pairs, but they should be regarded as hypothesis-generating rather than conclusive evidence. Dual-luciferase assays provide direct support for miRNA–target binding, yet they cannot fully reproduce the tissue-specific, developmental, or stress-dependent regulatory context *in vivo*. Conversely, agomir/antagomir- and RNAi-based interventions can link miRNA perturbation to resistance phenotypes, but their interpretation may be complicated by delivery efficiency, off-target effects, and the pleiotropic nature of miRNAs. Therefore, the most robust evidence should come from studies that combine transcriptomic screening, direct target validation, and organismal-level phenotypic assays under biologically relevant exposure conditions.

### 6.1. Research Paradigm for Mechanism Confirmation

Currently, a complete validation chain from omics screening to *in vitro* targeting confirmation and finally to *in vivo* phenotypic association is typically included in high-quality studies of miRNA–P450 regulatory mechanisms. Regarding *in vitro* confirmation, the dual-luciferase reporter assay is currently widely utilized to verify the direct physical binding between miRNAs and the 3′-UTRs of target genes. As mentioned in the aforementioned mechanistic studies on the brown planthopper and whitefly, this technique was employed as a core method, providing direct *in vitro* evidence for miRNA-mediated post-transcriptional repression [11,21]. Furthermore, to verify the necessity of the miRNA pathway in resistance formation at a macroscopic level, RNA interference (RNAi) technology is frequently utilized to systematically knock down key enzymes in the miRNA biogenesis and execution pathways *in vivo*, thereby observing phenotypic changes. For instance, in studies where *Dicer1*, *Drosha*, and *AGO1* were knocked down in *N. lugens* [25], and *Drosha*, *Dicer1*, and *Ago2A* were knocked down in *B. tabaci* [12], the blockade of these core enzymes directly led to alterations in insect susceptibility to insecticides. Indispensable *in vivo* evidence for the involvement of miRNAs in insecticide resistance regulation is provided by such experiments from the upstream pathway level. In addition to these pathway-level examples, direct manipulation of specific miRNA–P450 pairs has also provided functional evidence across different insect taxa. For example, manipulation of novel_85 in *N. lugens* altered *CYP6ER1* expression and nitenpyram susceptibility [21], while miR-276-3p manipulation in *B. tabaci* affected *CYP6CX3* expression and cyantraniliprole resistance [12]. Similar miRNA-specific validation has been reported for miR-190-5p–*CYP6K2* in *S. frugiperda* [20], miR-3037–*CYP6CY2* in *S. miscanthi* [26], and miR-4133-3p–*CYP4CJ1* in *A. gossypii* [38]. Together, these examples indicate that combining pathway-level disruption with specific miRNA–target manipulation can provide stronger evidence for causal miRNA–P450 regulatory relationships.

### 6.2. Technical Bottlenecks of In Vivo Functional Intervention and Off-Target Risks

Reproducing resistance or susceptibility phenotypes in live insects using reverse genetics approaches remains a major challenge in current mechanistic research. First, manipulating miRNA abundance at the *in vivo* level currently relies primarily on the microinjection or artificial feeding of miRNA agomirs or antagomirs. However, the efficacy of such delivery strategies is highly dependent on the anatomical structure and physiological characteristics of the insects [47,48]. For example, in hemipteran insects (such as *N. lugens*), small RNAs like miR-2703 can be successfully delivered via a non-invasive artificial feeding system (feeding assay), and phenotypic defects, along with increased mortality, can be observed at relatively low doses [49]. Conversely, for lepidopteran insects (such as the silkworm *B. mori*), due to their highly alkaline guts and the presence of highly active nucleases, microinjection techniques, which entail certain mechanical damage, are often required to achieve effective intervention against *in vivo* target molecules (e.g., miR-6497-5p) [28]. This instability of delivery efficiency across different insect orders increases the difficulty of *in vivo* phenotypic determination and constitutes a technical bottleneck that must be urgently overcome for future field applications based on small RNAs.

Second, due to the pleiotropic nature of miRNAs targeting multiple genes, potential off-target effects must be carefully evaluated when high concentrations of miRNA agomirs or dsRNAs are utilized. This potential risk has already been revealed by current molecular ecotoxicology studies. For instance, in safety assessments concerning the pollinating beneficial insect, the honey bee (*Apis mellifera*), transcriptomic analyses have indicated that exogenously introduced nucleic acid molecules (including the commonly used negative control dsGFP) can trigger non-specific gene expression fluctuations, interfering with hundreds of non-target genes associated with development and metabolism [50]. Similarly, it has been pointed out by bioinformatics predictions that potential off-target binding sites exist within the honey bee genome for certain insecticidal nucleic acid sequences designed against pests [51].

When evaluating the ecological risks of novel nucleic acid biopesticides on predatory natural enemies (e.g., lady beetles, *Coccinellidae*), the probability of homologous matching of long-sequence siRNAs generated from the degradation of exogenous dsRNAs in non-target species is typically required to be considered [52]. However, beyond this, off-target mechanisms similar to those of miRNAs also demand close attention. Studies have shown that small RNA fragments generated during the RNAi process can bind merely by relying on the “seed region” of 7–8 nucleotides at the 5′ end [53]. Such minor homology of short sequences is more prone to result in accidental targeting matches across species, which may subsequently interfere with the normal physiology and metabolism of non-target insects. Therefore, in future nucleic acid pesticide designs and ecological risk assessments [54], one must not be limited solely to the exact matching of long sequences; the cross-species off-target risks triggered by “one-to-many” targeted binding must also be systematically considered. Emerging genome-editing tools such as CRISPR/Cas9 further enable precise functional validation of miRNA–target interactions *in vivo* [55]. For example, CRISPR/Cas9-mediated editing of miRNA genomic loci or target-site sequences within the 3′-UTRs of candidate genes can help distinguish direct regulatory effects from indirect network-level responses [56,57]. Such approaches may provide stronger causal evidence than expression correlation or transient RNAi-based perturbation alone, although their application in non-model pest species still requires improvements in transformation efficiency, delivery systems, and off-target assessment [58].

## 7. Conclusions and Future Perspectives

### 7.1. Summary of Core Conclusions

Throughout the complex evolutionary history of insects, adaptive responses to toxic chemicals, including plant secondary metabolites and synthetic insecticides, have consistently represented a central topic in resistance research. Traditional studies on resistance mechanisms have predominantly focused on genomic mutations and transcription factor activation. However, extensive recent research has systematically revealed the critical role of miRNA-mediated post-transcriptional regulatory networks in this process.

Synthesizing existing studies, it is evident that the miRNA–P450 regulatory axis is not an isolated metabolic pathway but rather serves as a crucial node linking upstream environmental signals (e.g., toxicant stress and population density) and the endocrine system (e.g., juvenile hormone and ecdysone pathways) to fine-tune downstream detoxification networks. Through the inducible or constitutive downregulation of specific miRNAs, insects can achieve a “de-repression” effect on core P450 genes, rapidly upregulating detoxification enzyme expression to attain metabolic resistance and host adaptability. Simultaneously, this energetically costly post-transcriptional remodeling process may crosstalk with pleiotropic regulatory networks governing insect reproduction and development, thereby providing a potential molecular explanation for the fitness costs associated with resistance.

### 7.2. miRNA-Based Pest Management Strategies and Perspectives

In the face of the increasingly severe global challenge of chemical pesticide resistance, pest management strategies based on miRNA regulatory mechanisms have demonstrated significant application potential, offering novel insights for the development of nucleic acid biopesticides.

On the one hand, laboratory studies have demonstrated that restoring miRNA-mediated repression of detoxification enzymes through the injection or artificial feeding of miRNA agomirs has been shown under laboratory conditions to resensitize certain pest species, such as *N. lugens* and *S. frugiperda*, to specific insecticides [20,21]. Building on this mechanism, the future development of stable, highly penetrable nanoscale miRNA analogs is anticipated. When formulated with conventional chemical pesticides as “nucleic acid synergists”, this approach shows potential, although further validation is required for managing field populations exhibiting high-level resistance.

It is important to note, however, that the sole inhibition of detoxification enzymes cannot directly kill insects in a pesticide-free environment. Therefore, distinct from the synergist strategy, targets for developing independently lethal nucleic acid biopesticides must be essential genes that maintain core metabolic and physiological functions in insects. Accordingly, miRNAs that are uniquely expressed in target pest species and critically involved in essential life processes must be systematically identified. By delivering specific miRNA regulators (e.g., agomirs or antagomirs), the physiological homeostasis of the pest can be fundamentally disrupted, ultimately leading to mortality [6,15,16]. The principal challenge in developing such independent nucleic acid biopesticides lies in conducting exceptionally rigorous ecological safety assessments [50,51]. Given that highly conserved miRNAs carry a substantial risk of cross-species off-target toxicity to non-target organisms, including pollinators such as the honey bee (*A. mellifera*) and predatory natural enemies, future target screening must be strictly focused on miRNAs with minimal sequence conservation and high species specificity.

**Figure 3:**
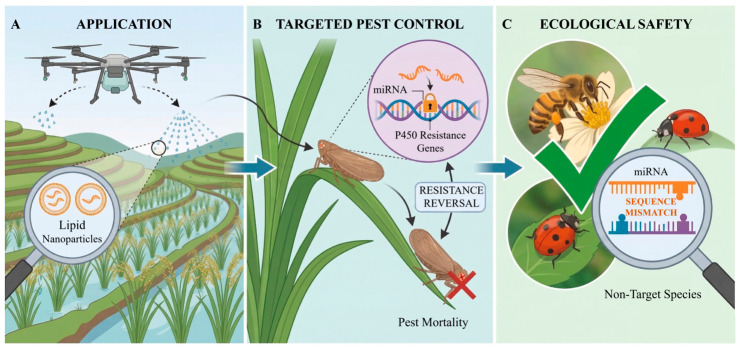
Conceptual framework for miRNA-based nucleic acid biopesticides and ecological safety assessment. miRNA analogs delivered using carriers such as lipid nanoparticles (LNPs) can modulate the expression of detoxification genes in target pests. Sequence specificity is required to minimize off-target interactions with non-target organisms, including pollinators and natural enemies. Ecological safety evaluation involves assessing sequence homology and potential cross-species effects.

Regardless of whether the objective is to develop nucleic acid synergists or independently lethal nucleic acid biopesticides, the integration of nanocarrier systems (such as lipid nanoparticles, or LNPs) or engineered symbiotic bacterial technologies to overcome the physiological barriers of insects and achieve targeted delivery of specific nucleic acid molecules is likely to represent an important research direction for advancing miRNA-based green biopesticides toward field application (Figure 3). A central practical obstacle, however, lies in delivering intact miRNA molecules across the insect’s physical and biochemical barriers to reach their intracellular targets. The insect cuticle, a hydrophobic and chemically cross-linked exoskeleton, severely restricts the penetration of topically applied nucleic acids, whereas the alkaline gut environment and abundant nucleases can rapidly degrade orally ingested RNA before it is taken up by target cells [47,54]. Even after cellular uptake, efficient endosomal escape and loading into the RNA-induced silencing complex (RISC) are required for miRNAs to exert post-transcriptional regulation. Consequently, naked miRNA or dsRNA generally shows poor and inconsistent bioavailability under field-relevant application routes [47,54]. Nanocarrier-based formulations, such as lipid nanoparticles and engineered peptide or polymer carriers, can protect RNA cargo from nuclease degradation and enhance transcuticular and transmembrane delivery, whereas symbiotic-bacteria-mediated systems may offer a sustained and less invasive route for *in vivo* RNA delivery [41,47,48,54,59]. Optimizing such delivery platforms to achieve reliable cuticle penetration and target-cell uptake, therefore, represents a prerequisite for translating laboratory-validated miRNA–P450 interventions into practical pest-management products. Despite substantial progress, several key questions remain unresolved. First, the stability and robustness of miRNA–P450 regulatory networks under complex and fluctuating field conditions are still poorly understood [3]. Second, it remains unclear whether a limited number of “master” miRNAs exert dominant control over resistance phenotypes or whether regulation is distributed across highly redundant networks [9]. Third, the extent to which miRNA-mediated resistance mechanisms can be sustainably manipulated without inducing rapid compensatory evolution requires further investigation [2]. Finally, balancing the efficacy of miRNA-based pest control strategies with ecological safety, particularly regarding off-target effects in non-target organisms, remains a critical challenge for future research [51,53].

## Figures and Tables

**Table 1:** Summary of miRNA-mediated post-transcriptional regulation of cytochrome P450s in insects. Bold terms in the first column indicate insect orders.

Insect Species	microRNA	Target P450	Stressor/Pesticide	Regulatory Mode and Effects	Validation Methods	Ref.
Hemiptera						
*Bemisia tabaci*	novel_miR-1517	*CYP6CM1*	Imidacloprid	Down-regulation mediates resistance	Dual-luciferase assay, miRNA mimic/inhibitor feeding, Western blotting assay, Bioassay	[11]
*B. tabaci*	miR-276-3p	*CYP6CX3*	Cyantraniliprole	Down-regulation mediates resistance	Dual-luciferase assay, miRNA mimic/inhibitor feeding, RNAi, Bioassay	[12]
*Nilaparvata lugens*	novel_85	*CYP6ER1*	Nitenpyram	Down-regulation mediates resistance	Dual-luciferase, miRNA mimic/inhibitor injection, RNAi, Bioassay	[21]
*N. lugens*	miR-210-5p	*CYP6ER1*	Clothianidin	Down-regulation mediates resistance	Dual-luciferase assay, miRNA agomir/antagomir injection, RNAi, Bioassay	[25]
*Sitobion miscanthi*	miR-3037	*CYP6CY2*	Imidacloprid	Down-regulation mediates resistance	Dual-luciferase assay, miRNA agomir/antagomir feeding, Bioassay	[26]
*Myzus persicae*	let-7, miR-100	*CYP6CY3*	Nicotine	Down-regulation mediates tolerance	Dual-luciferase assay, miRNA mimic/inhibitor feeding, RNAi	[37]
*Aphis gossypii*	miR-4133-3p	*CYP4CJ1*	Gossypol, Tannic acid	Down-regulation mediates tolerance	Dual-luciferase assay, miRNA agomir/antagomir feeding, RNAi	[38]
*Laodelphax striatellus*	PC-5p-3991_515	*CYP417A2*	Triflumezopyrim	Down-regulation mediates resistance	Dual-luciferase assay, miRNA antagomir/inhibitor feeding, FISH, RNAi, Bioassay	[39]
Lepidoptera						
*Spodoptera frugiperda*	miR-190-5p	*CYP6K2*	Chlorantraniliprole	Down-regulation mediates resistance	Dual-luciferase assay, miRNA antagomir/inhibitor injection, RNAi, Bioassay	[20]
*S. frugiperda*	miR-23a	*CYP9F1*	Chlorantraniliprole	Down-regulation mediates resistance	Dual-luciferase assay, miRNA agomir/antagomir injection, RNAi, Bioassay	[23]
*Bombyx mori*	miR-6497-5p	*CYP337A2*	Chlorfenapyr	Up-regulation prevents pro-drug activation	Dual-luciferase assay, miRNA agomir/antagomir injection, Bioassay	[28]
Diptera						
*Culex pipiens pallens*	miR-4448	*CYP4H31*	Deltamethrin	Down-regulation mediates resistance	Dual-luciferase assay, miRNA mimic/inhibitor injection, Bioassay, Western blotting assay	[13]
*Drosophila melanogaster*	miR-310s cluster	*Cyp6G1,* *Cyp6G2*	DDT	Down-regulation mediates resistance	Dual-luciferase assay, miRNA mimic injection, Bioassay	[22]

## Data Availability

No new data were created or analyzed in this study. Data sharing is not applicable to this article.

## Supplementary Materials

The following supporting information can be downloaded at https://www.mdpi.com/article/10.3390/genes17060698/s1, Figure S1: Three-level validation framework for miRNA–P450 regulatory interactions. Level I evidence is based on expression correlation analysis, typically derived from sRNA-seq or RNA-seq datasets, and is mainly used for candidate screening and hypothesis generation. Level II evidence involves direct target validation, most commonly through dual-luciferase reporter assays confirming physical binding between miRNAs and the 3′-UTRs of target P450 genes. Level III evidence provides in vivo functional validation through miRNA agomir/antagomir manipulation, RNAi-mediated pathway disruption, and insecticide susceptibility bioassays. Evidential strength and causal inference increase from Level I to Level III.
