# Supplementary material for: MicroRNA-Mediated Post-Transcriptional Regulation of Cytochrome P450s
Source: Genes (Basel). 2026 Jun 16;17(6):698. doi: 10.3390/genes17060698 (PMC13299453; doi:10.3390/genes17060698)
Supplement: Supplementary file 1 [file genes-17-00698-s001.zip › genes-4335185-supplementary.pdf]

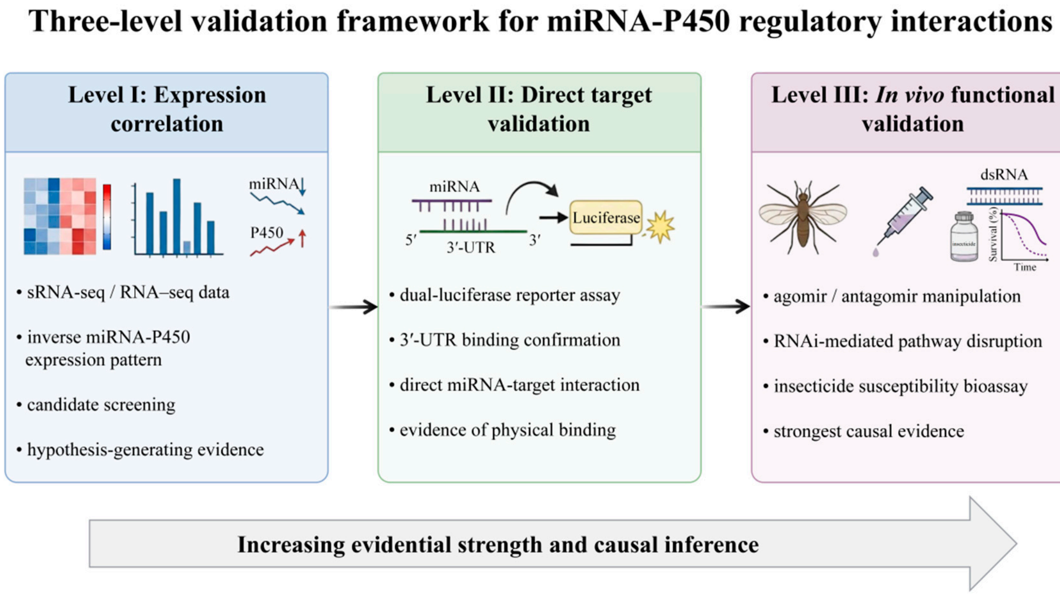

Figure S1. Three-level validation framework for miRNA–P450 regulatory interactions. Level I evidence is based on expression correlation analysis, typically derived from sRNA-seq or RNA-seq datasets, and is mainly used for candidate screening and hypothesis generation. Level II evidence involves direct target validation, most commonly through dual-luciferase reporter assays confirming physical binding between miRNAs and the 3′-UTRs of target P450 genes. Level III evidence provides *in vivo* functional validation through miRNA agomir/antagomir manipulation, RNAi-mediated pathway disruption, and insecticide susceptibility bioassays. Evidential strength and causal inference increase from Level I to Level III.
